# Post-activation potentiation enhancement induction strategies with different rest intervals on jump performance: a meta-analysis

**DOI:** 10.3389/fphys.2025.1696129

**Published:** 2026-01-15

**Authors:** Ying Zhou, Xiaoqin Zhang, Jian Wang

**Affiliations:** 1 Faculty of Sports Science, Ningbo University, Ningbo, China; 2 Department of Sports Science, Faculty of Physical Education, Sports, and Health, Srinakharinwirot University, Bangkok, Thailand; 3 Department of Physical Education, Ningbo University of Technology, Ningbo, China

**Keywords:** back squat, jumping ability, meta-analysis, post-activation potentiation enhancement, rest interval

## Abstract

**Objective:**

This meta-analysis aimed to systematically evaluate the effects of post-activation potentiation enhancement (PAPE) on jump performance and explore its optimal induction strategies.

**Methods:**

Randomized controlled trials (RCTs) investigating the influence of PAPE training on jump performance were retrieved from Web of Science, PubMed, Scopus, and EBSCO. Literature screening was conducted using the Cochrane Risk of Bias Tool. Quality assessment and statistical analyses were performed using RevMan 5.4 software, while sensitivity analysis and funnel plots were employed to evaluate result stability and publication bias.

**Results:**

A total of 22 RCTs involving 468 participants were included. The meta-analysis demonstrated that PAPE significantly improved jump performance [*SMD* = 1.36, 95% *CI* (0.89, 1.83), *P* < 0.0001]. Subgroup analysis indicated that exercise intensity might be a source of heterogeneity across studies.The largest effect sizes with statistical significance were observed in the following subgroups: exercise mode (Back squat) [*SMD* = 2.85, 95% *CI* (0.98, 4.73), *P* = 0.003], gender (Male) [*SMD* = 1.53, 95% *CI* (0.92, 2.14), *P* < 0.0001], outcome extracted (Counter movement jump) [*SMD* = 1.34, 95% *CI* (0.86, 1.81), *P* < 0.0001], exercise intensity (Moderate Intensity) [*SMD* = 2.46, 95% *CI* (1.71, 3.22), *P* < 0.0001], and rest interval (3–7 min) [*SMD* = 1.47, 95% *CI* (0.79, 2.14), *P* < 0.0001].

**Conclusion:**

PAPE may serve as a potentially effective strategy for enhancing jumping performance under appropriate conditions. In exercises aimed at improving jumping performance, back squats and medium-intensity induction appear to yield the most pronounced benefits. A 3–7 min recovery interval works best, though adjustments should be made based on individual exercise factors.

**Systematic Review Registration:**

http://inplasy.com, identifier INPLASY202430008.

## 1 Introduction

Jumping performance is a critical core athletic indicator in multiple competitive sports and recreational physical activities (Bazanov et al., 2019). Its level not only directly determines athletes’ competitive rankings and tactical execution efficiency but also serves as a key benchmark for evaluating lower limb explosive strength, neuromuscular coordination, and functional movement capacity (Wang et al., 2021). With the advancement of evidence-based sports training, optimizing jumping performance through non-invasive, time-efficient intervention strategies has become a frontier focus in sports physiology and exercise training science (Batista et al., 2011). Post-activation potentiation enhancement (PAPE) is defined as a transient physiological phenomenon wherein short-duration, high-intensity preconditioning stimuli induce acute improvements in subsequent explosive motor performance (Masagca, 2024). Due to its advantages of no additional training load burden and rapid neuromuscular optimization, PAPE has emerged as a promising strategy for enhancing jumping performance, providing crucial theoretical support for designing pre-competition warm-up protocols and in-season training microcycles in jumping-dominant sports (Yu et al., 2024; Chen et al., 2023).

The physiological mechanisms underlying PAPE primarily involve enhanced calcium ion release from the sarcoplasmic reticulum (Ishii et al., 2023), improved actin-myosin cross-bridge cycling efficiency (Mckiel et al., 2024), upregulated α-motor neuron excitability (Dos et al., 2023), and increased muscle-tendon unit stiffness (Wang et al., 2023). However, the magnitude and sustainability of PAPE effects are highly dependent on the design of preconditioning stimuli (Liu et al., 2024). Despite extensive empirical research on PAPE-induced jumping performance improvements (Cui et al., 2024), existing studies exhibit substantial heterogeneity in intervention outcomes, primarily attributed to inconsistent manipulation of key variables: load intensity (30%–100% 1RM), load volume (3–10 repetitions), recovery time (2–20 min), and subject characteristics (e.g., training experience, muscle fiber type distribution) (Yu et al., 2024). Current mainstream PAPE induction methods include loaded resistance exercises (e.g., back squats, Romanian deadlifts), explosive plyometric movements (e.g., medicine ball throws, drop jumps) (Li et al., 2024), and isometric contractions (e.g., static squat holds, hip thrusts) (Terbalyan et al., 2025). These methods differ significantly in neuromuscular activation patterns (e.g., rate of force development, RFD; electromyographic amplitude, EMG) and metabolic responses (e.g., lactate accumulation, oxygen consumption) (Beattie et al., 2014), but their differential effects on specific jumping metrics (vertical jump height, VJ; counter movement jump, CMJ; peak power) remain poorly characterized.

Scientific warm-up protocols are well-documented to mitigate injury risk and enhance acute athletic performance (Zhou et al., 2024). Integrating PAPE into warm-up procedures to shorten pre-competition preparation time and optimize high-intensity exercise capacity has important theoretical and practical implications for competitive sports (Ewertowska et al., 2023). However, three critical research gaps persist in the current literature: (1) no consensus has been reached on which PAPE induction method yields the most robust and consistent improvements in jumping performance, particularly across different jumping types (e.g., CMJ); (2) the interaction between induction method and recovery time—i.e., whether different methods require distinct recovery windows to exert optimal PAPE effects—has not been systematically investigated; and (3) few studies have quantified the influence of individual characteristics (e.g., baseline strength level) on PAPE responsiveness across different induction methods.

To address these gaps, this study aims to: (1) Systematically compare the acute effects of different PAPE induction methods across varying rest interval durations on the explosive jumping performance of healthy young adults, including CMJ height, standing long jump distance, and vertical jump peak power; (2) identify the optimal recovery time window for each induction method to maximize jumping performance improvements; and (3) explore the moderating role of baseline lower limb strength on PAPE responsiveness. The findings of this study are expected to provide evidence-based theoretical support and practical guidelines for athletes, coaches, and sports scientists to select individualized, efficient PAPE induction strategies, thereby advancing the scientificization of training and competition in jumping-related sports.

## 2 Materials and methods

### 2.1 Search strategy

A systematic literature search was performed across four electronic databases: Web of Science, PubMed, Scopus, and EBSCO, following the Preferred Reporting Items for Systematic Reviews and Meta-Analyses (PRISMA) guidelines (Schmidt et al., 2019). The search period covered the inception of each database to 1 May 2025, yielding a total of 1909 initial records. The PICO (Population, Intervention, Comparison, Outcome) framework was strictly applied to design the search strategy: Population = healthy individuals; Intervention = PAPE induction methods; Comparison = alternative intervention or no intervention; Outcome = jump performance indicators. English search terms were optimized for consistency and comprehensiveness as follows: (“PAPE” OR “Post-activation potentiation” OR “Post activation potentiation”) AND (“Jump performance” OR “Jump” OR “Vertical jump” OR “Jump height” OR “CMJ” OR “Countermovement jump” OR “Squat jump”) AND (“Randomized controlled trial” OR “RCT”).

### 2.2 Inclusion and exclusion criteria

#### 2.2.1 Inclusion criteria

Study Design: Published studies investigating the effects of post-activation potentiation enhancement induction methods on jump performance indicators.

Participants: Healthy individuals aged ≤45 years (all were undergraduate students or athletes specializing in football, track and field, and other sports).

Intervention: The experimental group must receive PAPE-related exercises with clear documentation of exercise type, repetitions, sets, and intensity.

Control Group: The control group should either undergo alternative training methods or no training intervention.

Outcome Measures: Studies must report quantitative data on jump height (cm), including but not limited to counter movement jump (CMJ) height, squat jump height, and vertical jump height.

Accessibility: Full-text articles published in peer-reviewed journals.

#### 2.2.2 Exclusion criteria

Study Design: Non-randomized controlled trials, observational studies, review articles, or case reports.

Unrelated Interventions: Studies that do not involve post-activation potentiation enhancement research or focus on non-jump performance indicators.

Ineligible Populations: Studies involving participants with chronic diseases or animal models.

Insufficient Outcomes: Research lacking quantitative data on jump performance.

Duplicate or Inaccessible Data: Duplicate publications, studies with incomplete data, or unavailable full texts.

### 2.3 Data extraction

**FIGURE 1:**
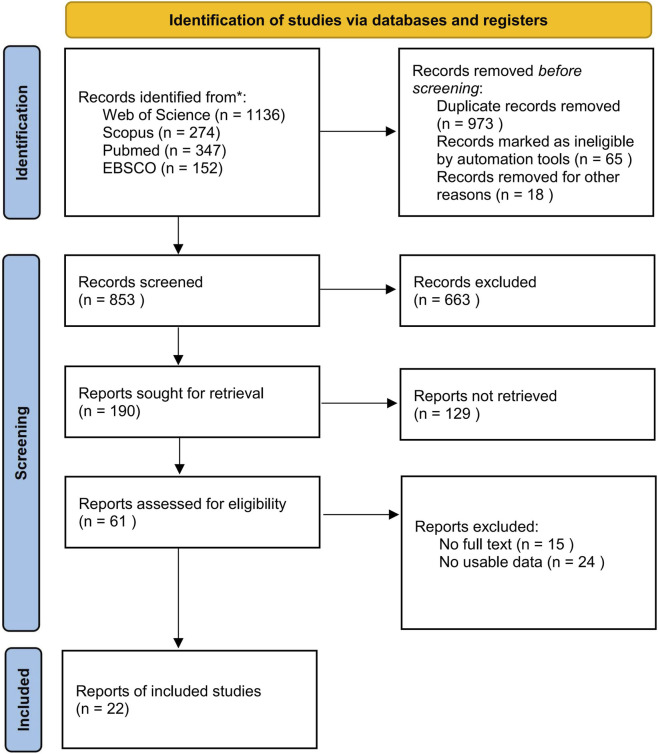
Flow diagram of literature selection.

All retrieved records were imported into EndNote software for de-duplication and management. Two independent researchers (J.W. and Y.Z.) screened the titles, abstracts, and full texts sequentially based on the predefined inclusion and exclusion criteria. Disagreements were resolved through discussion, negotiation, or adjudication by a third researcher. The process of literature screening and inclusion is illustrated in Figure 1. Ultimately, a total of 22 articles were included in the analysis.

Two researchers extracted data from the eligible literature using a customized data extraction form, which primarily included the following information:

1. General information: First author and year of publication.
2. Sample characteristics: Study participants, gender, age, and sample size of the experimental group.
3. Experimental characteristics: Intervention protocols for the experimental group, including training methods, number of sets, frequency, and training intensity.
4. Outcome measure: Jump height.

### 2.4 Statistical analysis

Statistical analyses were performed using RevMan 5.4 software (Page et al., 2021). The standardized mean difference (*SMD*) and 95% confidence interval (95% *CI*) were selected as the effect sizes for pooling combined effect magnitudes. The Cochrane Risk of Bias Assessment Tool was employed to evaluate the quality of the included studies (Higgins and Altman, 2007). Prior to conducting the comprehensive meta-analysis, a heterogeneity test was performed first. Homogeneity testing (Q-test, with a significance level of α = 0.1) was used for the heterogeneity assessment. The value of *I* ^2^ ranges from 0% to 100%. When *I* ^2^ > 50% and *p* < *α*, significant heterogeneity was considered to exist, and a random-effects model was selected for the meta-analysis. In contrast, a fixed-effects model was adopted. Subgroup analysis was conducted to address heterogeneity, and STATA 16.0 software was used for sensitivity analysis to examine the stability of the results. A funnel plot was utilized to verify the presence of publication bias.

## 3 Results

### 3.1 Study characteristics

A total of 22 publications were included in this study. All of these publications were randomized controlled trials (RCTs), involving 468 subjects of mixed genders, with ages ranging from 11 to 43 years. The basic characteristics of the included studies are presented in Table 1.

**TABLE 1:** Characteristic of studies included in systematic review and meta-analysis.

Study	Country	Age (years)	Sample	Type of PAPE (Interval)	Sets/Repetitions/Intensity	Jumping results
Sirieiro et al. (2021)	Brazil	17.3 ± 5.4	16M	Half squat (4 min)	1/5/65%1RM	CMJ↑
Doma et al. (2020)	Australia	22.9 ± 5.0	18M	Single leg lunges + high knee and hip kicks each (Immediate)	3/8/self weight	SJ NS
Kobal et al. (2019)	Brazil	25.42 ± 3.58	18M	Half squat (4 min)	1/6/60%1RM	CMJ↑
Piper et al. (2020)	United States	20 ± 2	13M, 3F	Back squat (2 min)	3/5/87%1RM	CMJ↑
Masel et al. (2023)	Poland	22.9 ± 2.1	15M	trap bar deadlift with elastic band (Immediate)	1/3/80%1RM	VJ↑
Timon et al. (2019)	Spain	21.8 ± 2.7	9M, 7F	Half squat (1 min)	3/6/Pmax	VJ NS
Spieszny et al. (2022)	Poland	19 ± 2	31M	Maximum isometric back squats (5 min)	3/3 s/max voluntary contraction	SJ NS
Sun et al. (2024)	China	22.3 ± 1.3	16M	Squat (1 min)	8/8/80%1RM	SJ↑ CMJ↑
Chaves et al. (2024)	Portugal	22.03 ± 2.80	25F	Maximum isometric back squats (2 min)	3/10 s/max voluntary contraction	CMJ↑
Yang et al. (2024)	China	18.25 ± 2.55	20F	Squat Jump (4 min)	1/6/self weight	CMJ↑
Gervasi et al. (2018)	Italy	26.6 ± 6	7M	Running (10 min)	1/40 min/Lt (4.0 mmol/L)	CMJ↑
Karakoç et al. (2025)	Türkiye	21.41 ± 1.37	17M	Squat (2 min)	1/6/80%1RM	CMJ↑
Beato et al. (2019)	Britain	22 ± 2	10M	Half squat (1 min)	3/6/Pmax	CMJ↑
Moré et al. (2023)	Brazil	34.1 ± 9.4	18M	Running (8 min)	1/11.4 ± 2.3 min/97.2HRmax	CMJ↑
Sañudo et al. (2020)	Spain	23.5 ± 5.3	28M	Squat (2 min)	1/3/90%1RM	CMJ↑
Ouergui et al. (2022)	Tunisia	16 ± 1	14M, 13F	Squat jump (4 min)	3/5/self weight	CMJ↑
Perenc et al. (2025)	Poland	22.5 ± 2.3	14M	Back squat (4 min)	2/2/55%1RM	CMJ↑
Hammami et al. (2022)	Tunisia	12 ± 1	30M	Hurdle jump (5 min)	3/6/30 cm	CMJ↑
Koźlenia and Domaradzki (2023a)	Poland	21.2 ± 1.85	30M, 15F	Isometric back squats (4 min)	3/4 s/70%1RM	CMJ↑
Guerra et al. (2018)	Brazil	23.83 ± 5.06	12M	Hurdle jump + Sled towing (8 min)	3/5/50 cm 3/20 m/15% self weight	CMJ NS
Xie et al. (2022)	China	20.63 ± 1.32	12M	Half squat (3 min)	3/6/60%1RM	CMJ↑
Koźlenia and Domaradzki (2023b)	Poland	21.07 ± 1.83	41F	Isometric back squat (4 min)	3/4 s/70%1RM	CMJ↑

M, male; F, female; RM, repetition maximum; NS, no statistical significance; ↑ represents a significant increase; CMJ, counter movement jump; Pmax, peak power; SJ, squat jump; VJ, vertical jump; Lt, lactate threshold.

### 3.2 Study quality assessment

**FIGURE 2:**
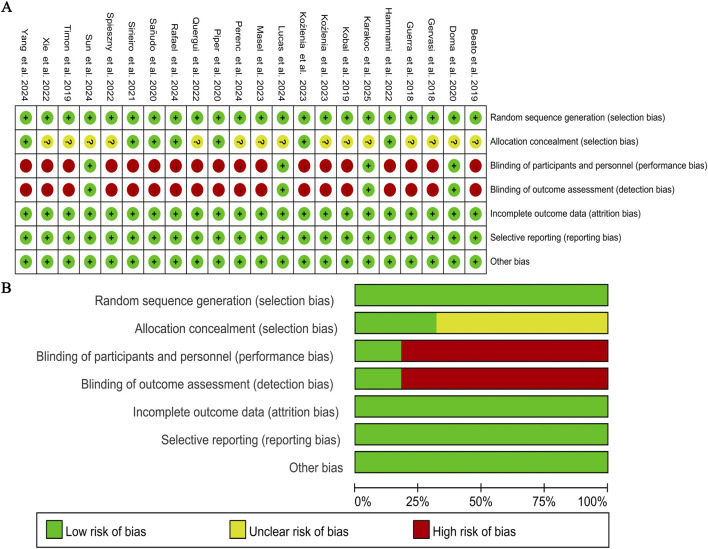
Methodological quality graph and summary of the included studies: **(A)** Risk of bias summary; **(B)** Risk of bias graph.

The methodological quality of the included RCTs was independently evaluated by two researchers (J.W. and Y.Z.) using the Cochrane Risk of Bias tool. Review Manager 5.4 software was used to assess seven key domains: random sequence generation, allocation concealment, blinding of participants, blinding of outcome assessment, incomplete outcome data, selective reporting, and other sources of bias (Figures 2A,B). Among the included studies, 15 failed to clearly state whether allocation staff strictly followed the random allocation process. Additionally, 18 had a high risk of bias in blinding, as participants signed informed consent forms before the experiment.

### 3.3 Jumping ability

**FIGURE 3:**
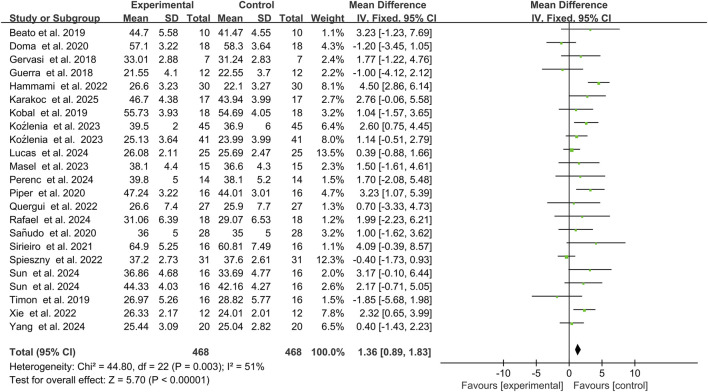
Effect of PAPE on jumping ability.

A total of 22 studies reported the relationship between PAPE induction methods and jumping performance, involving 468 subjects in aggregate. Heterogeneity testing indicated *I* ^2^ = 51% > 50%, and the *Q*-test yielded *p* = 0.003, suggesting substantial heterogeneity among the included studies. A random-effects model was therefore applied for meta-analysis (Figure 3). The results showed a combined effect size *SMD* = 1.36, which was statistically significant (*Z* = 5.70, *P* < 0.0001), indicating that appropriate PAPE induction protocols can improve subjects’ jumping performance compared with the control group.

### 3.4 Subgroup analysis

Based on the heterogeneity characteristics observed in this study, we speculate that the heterogeneity may originate from exercise mode, gender, outcome extracted, exercise intensity, and rest interval (Table 2).

**TABLE 2:** Subgroup analysis of the effects of PAPE on jumping ability.

Research features	Subgroup standard	Study (sample)	SMD	95%*CI*	*P*	*I* ^2^ (%)	*P* (Heterogeneity)
Exercise mode	Half squat	5 (72)	1.82	0.60, 3.03	0.004**	28	0.23
	Back squat	2 (30)	2.85	0.98, 4.73	0.003**	0	0.49
	Isometric back	4 (142)	0.65	−0.09, 1.38	0.08	58	0.07
	Squat	4 (77)	2.16	0.73, 3.60	0.003**	0	0.73
	Running	2 (25)	1.84	−0.60, 4.28	0.14	0	0.93
	Jumping	4 (89)	2.07	0.98, 3.17	0.0002***	81	0.001**
	Compound exercise	2 (33)	−0.28	−2.10, 1.54	0.77	47	0.17
Gender	Male	16 (278)	1.53	0.92, 2.14	<0.0001***	56	0.004**
	Female	3 (90)	0.93	0.02, 1.84	0.05	52	0.13
	Mixed	4 (100)	1.45	0.26, 2.63	0.02*	48	0.13
Outcome extracted	CMJ	22 (452)	1.34	0.86, 1.81	<0.0001***	53	0.002**
	SJ	3 (65)	−0.23	−1.29, 0.83	0.67	42	0.18
	VJ	2 (31)	0.17	−2.25, 2.58	0.89	44	0.18
Exercise intensity	Low Intensity	4 (77)	−0.27	−1.50, 0.96	0.67	0	0.66
	Moderate Intensity	8 (183)	2.46	1.71, 3.22	<0.0001***	33	0.17
	High Intensity	11 (208)	0.95	0.27, 1.63	0.006**	39	0.09
Rest interval	≤3 min	11 (189)	1.32	0.63, 2.01	0.0002***	42	0.07
	3∼7 min	9 (242)	1.47	0.79, 2.14	<0.0001***	68	0.001**
	≥8 min	3 (37)	0.77	−1.16, 2.69	0.43	0	0.37

*, *P* < 0.05; **, *P* < 0.05; ***, *P* < 0.05; CMJ, counter movement jump; SJ, squat jump; VJ, vertical jump.

In the exercise mode subgroup, the Back squat, Squat, and Running groups all showed homogeneity (*I* ^2^ = 0%), while compared with the overall combined effect size (*I* ^2^ = 51%), the Isometric back (*I* ^2^ = 58%) and Jumping (*I* ^2^ = 81%) subgroups exhibited higher heterogeneity, indicating substantial heterogeneity among studies within these two subgroups. Back squat yielded the largest effect size, which was statistically significant (*SMD* = 2.85, *P* = 0.003 < 0.01), suggesting that this activation method is more conducive to improving jumping performance.

In the gender subgroup, the heterogeneity values for the three groups were 56%, 52%, and 48%, respectively. Compared with the overall combined effect (*I* ^2^ = 51%), both the male and female subgroups demonstrated higher heterogeneity. The male subgroup showed the largest effect size (*SMD* = 1.53, *P* < 0.0001), indicating that males may be more responsive to PAPE induction methods aimed at enhancing jumping performance.

In the outcome extracted subgroup, the heterogeneity values for the three groups were 53%, 42%, and 44%, respectively. Compared with the overall combined effect (*I* ^2^ = 51%), the CMJ subgroup exhibited higher heterogeneity and also demonstrated the largest effect size (*SMD* = 1.34, *P* = 0.003 < 0.01), indicating that PAPE induction exercises can significantly improve subjects’ CMJ performance.

In the exercise intensity subgroup, the heterogeneity values for the low-, medium-, and high-intensity groups were 0%, 33%, and 39%, respectively, all of which were lower than the overall combined effect (*I* ^2^ = 51%). This suggests that varying exercise intensities may be one of the sources of heterogeneity. The medium-intensity group yielded the largest effect size, which was statistically significant (*SMD* = 2.46, *P* < 0.0001), indicating that PAPE induced by medium-intensity exercise can significantly enhance subjects’ jumping performance.

In the rest interval subgroup, the heterogeneity values for the three groups were 42%, 68%, and 0%, respectively. Compared with the overall combined effect (*I* ^2^ = 51%), the 3∼7 min subgroup showed higher heterogeneity and also produced the largest effect size (*SMD* = 1.34, *P* = 0.003 < 0.01), suggesting that a rest interval of 3∼7 min following PAPE induction can significantly improve subjects’ jumping performance.

### 3.5 Sensitivity analysis

Sensitivity analysis was conducted using the leave-one-out method to evaluate the heterogeneity of the included studies.

As shown in Table 3, the pooled effect size of PAPE on jumping performance was [*SMD* = 1.36, 95% *CI* (0.89, 1.83), *p* < 0.0001]. After sequentially removing individual studies, the pooled SMD ranged from 1.08 to 1.61, and the heterogeneity index I^2^ varied between 29% and 53%. Specifically, after excluding the studies by Doma et al. (2020), Hammami et al. (2022), and Spieszny et al. (2022), the heterogeneity decreased to 47%, 29%, and 43%, respectively. All results remained statistically significant (*p* < 0.01). No single study threatened the overall meta-analysis results, indicating that the findings of this study are relatively stable.

**TABLE 3:** Combined effects of jumping ability after excluding individual studies.

Study	*SMD*	95%*CI*	*P*(Merge effect)	*I* ^2^(%)
Beato et al. (2019)	1.34	0.87, 1.81	<0.0001	52
Doma et al. (2020)	1.47	1.00, 1.95	<0.0001	47
Gervasi et al. (2018)	1.35	0.87, 1.82	<0.0001	53
Guerra et al. (2018)	1.41	0.94, 1.88	<0.0001	51
Hammami et al. (2022)	1.08	0.60, 1.57	<0.0001	29
Karakoç et al. (2025)	1.32	0.84, 1.79	<0.0001	52
Kobal et al. (2019)	1.37	0.89, 1.84	<0.0001	53
Koźlenia and Domaradzki (2023a)	1.27	0.79, 1.76	<0.0001	51
Koźlenia and Domaradzki (2023b)	1.38	0.89, 1.86	<0.0001	53
Chaves et al. (2024)	1.51	1.01, 2.01	<0.0001	50
Masel et al. (2023)	1.35	0.88, 1.83	<0.0001	53
Perenc et al. (2025)	1.35	0.88, 1.82	<0.0001	53
Spieszny et al. (2022)	1.27	0.79, 1.74	<0.0001	50
Ouergui et al. (2022)	1.37	0.90, 1.84	<0.0001	53
Moré et al. (2023)	1.35	0.88, 1.82	<0.0001	53
Sañudo et al. (2020)	1.37	0.90, 1.84	<0.0001	53
Sirieiro et al. (2021)	1.33	0.86, 1.80	<0.0001	52
Spieszny et al. (2022)	1.61	1.11, 2.10	<0.0001	43
Sun et al. (2024)	1.32	0.85, 1.79	<0.0001	52
Sun et al. (2024)	1.34	0.86, 1.81	<0.0001	53
Timon et al. (2019)	1.41	0.94, 1.88	<0.0001	50
Xie et al. (2022)	1.28	0.79, 1.76	<0.0001	52
Yang et al. (2024)	1.42	0.94, 1.91	<0.0001	52
Overall	1.36	0.89, 1.83	<0.0001	51

The same literature name refers to different research results included in the same literature.

### 3.6 Publication bias

**FIGURE 4:**
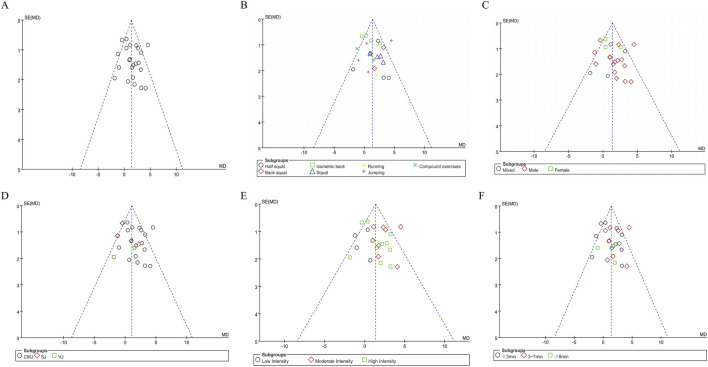
Funnel plots of jumping ability: **(A)** Combine funnel chart; **(B)** Exercise mode; **(C)** Gender; **(D)** Outcome extracted; **(E)** Exercise intensity; **(F)** Rest interval.

This study constructed funnel plots for each subgroup to assess potential publication bias. As shown in Figure 4, the funnel plots exhibited an approximately symmetrical shape. Egger’s test was further conducted on these funnel plots, and the results showed that the p-values for all subgroups were greater than 0.05, indicating no significant publication bias among the included studies.

## 4 Discussion

### 4.1 The effect of PAPE on jumping ability

This study investigated the effects of different PAPE induction methods on jumping performance through a meta-analysis, incorporating a total of 22 studies involving 468 subjects. Random-effects model analysis revealed a pooled effect size of *SMD* = 1.36 (*p* < 0.0001), indicating that PAPE induction can significantly improve jumping performance. The enhancement of jumping ability primarily relies on neural adaptive changes, optimization of muscular mechanical properties, and improved energy utilization efficiency (Aeles et al., 2018). PAPE acts through multiple pathways on these mechanisms to further enhance explosive performance.

From the perspective of neuromuscular function, jumping performance is closely related to neural drive capacity, motor unit recruitment rate and synchronization, as well as muscle fiber contraction characteristics (Odetti et al., 2015). PAPE induction activates high-threshold motor neurons through high-intensity preconditioning stimuli, increasing spinal excitability and descending drive signals, thereby optimizing muscle activation efficiency (Hamada et al., 2000)^.^ Studies have shown that phosphorylation of myosin regulatory light chains can enhance calcium ion (Ca^2+^) sensitivity within the sarcoplasm, accelerate cross-bridge cycling rate, and consequently improve the RFD—a key mechanical factor determining jump height (Daniel et al., 2023). Research also indicates that muscles under PAPE conditions can more effectively utilize elastic potential energy, enhancing stretch-shortening cycle (SSC) efficiency (Al et al., 2025), which is particularly critical for continuous and reactive jumping performance.

Furthermore, PAPE induction exhibits a selective activation effect on type II muscle fibers. Following high-intensity conditioning contractions, the recruitment threshold of fast-twitch fibers is temporarily lowered, making them more readily mobilized in subsequent explosive activities, thereby contributing to greater power and force output (Monteiro-Oliveira et al., 2022). Appropriate PAPE induction can optimize signal transduction at the neuromuscular junction, increasing the discharge frequency of motor units per unit time, which significantly improves jump height and take-off velocity (Pourmoghaddam et al., 2016).

The results of this study demonstrate a high degree of consistency, indicating that PAPE, as a training strategy, possesses strong generalizability and can be applied to different populations and various sports contexts. Future research should focus on clarifying the interactions between different induction protocols and individual characteristics (such as muscle fiber type, training experience, and genetic background), and further utilize techniques such as EMG and transcranial magnetic stimulation (TMS) to elucidate the central and peripheral mechanisms of PAPE.

### 4.2 Moderating factors of PAPE in enhancing jumping performance

The PAPE effect is regulated by multiple factors. For instance, although both loaded back squats and drop jumps can be used as PAPE induction methods, their neural adaptation patterns and fatigue-potentiation balance points differ, potentially leading to varying effects on different types of jumps such as CMJ, SJ, or Drop Jump (Ohta et al., 2013). Furthermore, the optimal rest interval duration is often influenced by an individual’s strength level and recovery capacity: untrained individuals may experience PAPE benefits masked by fatigue accumulation, whereas elite athletes can more effectively utilize shorter time windows to achieve neuromuscular enhancement (Boullosa et al., 2018).

This study identified substantial heterogeneity (*I* ^2^ = 51%, *p* = 0.003), indicating that the PAPE effect is modulated by multiple factors. To further investigate this, subgroup analyses were conducted based on exercise mode, gender, outcome extracted, exercise intensity, and rest interval, thereby providing deeper insights into the influencing factors of the PAPE effect.

#### 4.2.1 Exercise mode

From the perspective of induction methods, the effect sizes produced by different PAPE induction protocols exhibit distinct differences. Maximal voluntary contractions (MVC) and heavy resistance training (>85% 1RM) typically elicit stronger neural adaptations and myosin light chain phosphorylation, thereby demonstrating more prominent effects in enhancing vertical jump performance such as CMJ and SJ (Garbisu-Hualde and Santos-Concejero, 2021). In contrast, ballistic training (e.g., drop jumps, loaded jumps) offers unique advantages in improving stretch-shortening cycle (SSC) efficiency and reactive jump capacity due to its closer resemblance to sport-specific movement patterns (Wilk et al., 2020). This finding aligns with the “movement specificity principle” proposed by Wilk et al., which suggests that the transfer effect of PAPE is more significant when the induction exercise closely matches the biomechanical and neuromuscular control patterns of the target movement (Chambon et al., 2010).

#### 4.2.2 Gender

The effect size for males (*SMD* = 1.41) was slightly higher than that for females (*SMD* = 1.19), though the between-group difference did not reach statistical significance. This trend may be related to muscle volume, hormonal environment, and muscle fiber type composition. Existing studies indicate that individuals with higher androgen levels typically possess a greater proportion of fast-twitch fibers and stronger neural drive capacity, which may contribute to more effective expression of power gains during PAPE induction (Daniel et al., 2023). Nevertheless, females can still achieve significant benefits through appropriately designed PAPE protocols, demonstrating that PAPE is an effective strategy applicable to both genders. However, potential influences such as hormonal fluctuations and strength levels should be considered when designing individualized programs.

#### 4.2.3 Outcome extracted

Different jump test metrics exhibit varying sensitivity to PAPE. The highest pooled effect size was observed for CMJ (*SMD* = 1.48), while Drop Jump showed a relatively lower response (*SMD* = 1.21). CMJ performance is highly dependent on voluntary force production capacity and the RFD, making it more sensitive to enhancements in neural drive. In contrast, Drop Jump relies more on SSC efficiency and tendon stiffness, and its response may be influenced by the degree of specificity between the induction method and the sport-specific movement pattern (Ratamess et al., 2009).

CMJ jump height, as a valid metric for assessing the PAPE effect, is commonly quantified using the following formula (Cleary and Cook, 2020):

$$\text{PAP}\left(\%\right)=\left[\frac{\text{post CMJ height }\left(\text{cm}\right)}{\text{pre CMJ height }\left(\text{cm}\right)}\times 100\right]$$ (1)

A value greater than 100 indicates the presence of PAPE. Among the studies incorporating CMJ jump height, all 22 studies reported positive effect sizes (*SMD* = 1.34). Thus, this study further validates the optimizing effect of PAPE induction on CMJ performance, supporting the view proposed.

#### 4.2.4 Exercise intensity

Based on physiological and loading indicators such as percentage of maximum heart rate (%HRmax), blood lactate concentration (mmol/L) (Weigelin and Jessen, 1981), and percentage of one-repetition maximum (%1RM) (Karvonen and Vuorimaa, 1988), this study categorized exercise intensity into low-, medium-, and high-intensity subgroups (Table 4). Analysis revealed that the heterogeneity values for the low-, medium-, and high-intensity subgroups were 0%, 33%, and 39%, respectively, all lower than the overall heterogeneity (*I* ^2^ = 51%), indicating that exercise intensity is a significant moderating factor contributing to the variability in results across studies. Notably, the medium-intensity subgroup demonstrated the largest effect size (*SMD* = 2.46, *p* < 0.0001), significantly outperforming both the low- and high-intensity subgroups. This suggests that PAPE induction implemented within this intensity range is most effective for enhancing jumping performance.

**TABLE 4:** Exercise intensity classification standards.

Intensity level	Resistance load (%1RM)	BLA (mmol/L)	Cardiovascular response (%HRmax)
Low Intensity	<60%	<2 mmol/L	<70%
Moderate Intensity	60∼80%	2∼4 mmol/L	70∼85%
High Intensity	>80%	>4 mmol/L	>85%

On one hand, this intensity (e.g., 80% 1RM) is sufficient to activate high-threshold type II muscle fibers, inducing adequate myosin light chain phosphorylation (Ayesta et al., 2006), which enhances calcium ion sensitivity and cross-bridge cycling rate, thereby providing the necessary neurophysiological foundation for explosive performance (Julian et al., 2021). On the other hand, compared to higher intensities (>85% 1RM), medium-load induction generates substantially less metabolic stress and central fatigue, allowing fatigue components to dissipate more rapidly. Consequently, the PAPE effect can be more fully expressed during the recovery period.

Although higher-intensity loads can theoretically induce stronger neural excitation and physiological stress, they simultaneously lead to more pronounced fatigue accumulation, often resulting in the “fatigue-masking effect” dominating and thereby reducing the net benefit of PAPE (Sabag et al., 2018). In contrast, low-intensity stimuli (<70% 1RM) are unable to effectively recruit fast-twitch muscle fibers or trigger sufficient molecular signaling responses, making it difficult to produce meaningful potentiation effects.

Therefore, in practice, it is recommended to use medium intensity (75%–85% 1RM) as the preferred range for PAPE induction. At the same time, individual adjustments should be made based on population characteristics and sport-specific demands, supplemented by real-time monitoring and personalized regulation using physiological indicators such as blood lactate and heart rate variability. This approach aims to maximize the enhancement effect of PAPE on jumping performance.

#### 4.2.5 Rest interval

Rest interval is a critical temporal factor modulating PAPE benefits, directly influencing the balance between fatigue recovery and potentiation effects. This study indicates that 3–7 min represents the overall optimal recovery window (*SMD* = 1.47). Shorter intervals (<3 min) may allow fatigue to dominate, resulting in non-significant or even diminished performance improvements. Intervals exceeding 5 min lead to a gradual decline in neural excitability and calcium sensitivity, causing the PAPE effect to diminish. These findings are highly consistent with the previously proposed “fatigue enhancement two-phase theory” (Tillin and Bishop, 2009).

**FIGURE 5:**
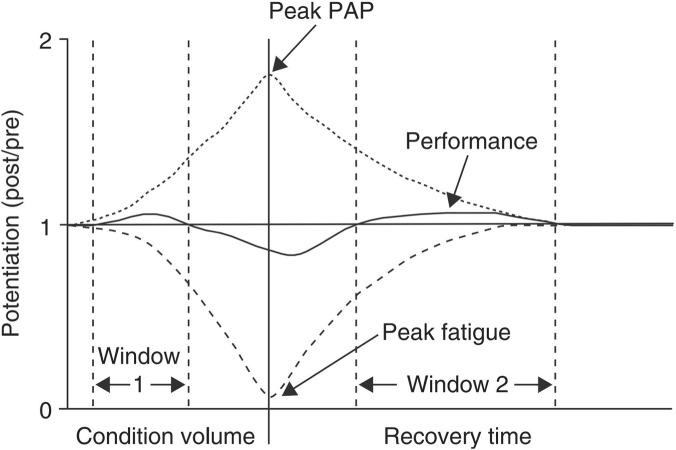
PAPE and fatigue model (Tillin and Bishop, 2009).

Under appropriate induction intensity, the PAPE effect may exhibit two distinct windows: the first occurs immediately to 2 min post-high-intensity loading, when neural excitability is elevated but fatigue has not fully dissipated (Figure 5). Subsequently, during the 3–7 min period, as the phosphagen system recovers and metabolic byproducts are cleared, fatigue decreases rapidly, allowing the PAPE effect to again dominate and form a more stable and pronounced second window of enhancement.

It is noteworthy that the optimal rest interval also varies with individual training status and induction load. Elite athletes, owing to faster phosphagen resynthesis and superior neural inhibitory control, may enter the PAPE-dominant phase within shorter intervals (e.g., 2–3 min) (Dobbs et al., 2018). In contrast, less-trained individuals or those using very high-intensity induction (e.g., >90% 1RM) often require longer intervals (4–6 min) to maximize the potentiation effect due to greater accumulation of fatigue metabolites (Daniel et al., 2023). Therefore, in practical training, rest intervals should be individualized based on both personal characteristics and induction intensity. Real-time monitoring of vertical jump performance or use of portable EMG devices to identify the optimal force production window can further enable precise exploitation of the PAPE effect.

### 4.3 Heterogeneity and methodological bias: key findings and implications

Moderate heterogeneity (I^2^ = 51%, p = 0.003) was observed for the effect of PAPE on jumping performance via random-effects modeling, partially explained by exercise intensity (0%–39% subgroup heterogeneity; moderate intensity: SMD = 2.46), movement mode (consistent with the specificity principle), rest interval (optimal 3–7 min), and outcome measures (CMJ more sensitive than SJ). Unaccounted variability may relate to unreported factors (e.g., training status, muscle fiber type) and inconsistent intervention protocols (e.g., repetition counts, movement standardization). Egger’s tests, and trim-and-fill analysis while sensitivity analysis validated result robustness. Heterogeneity highlights the necessity of personalized PAPE protocols, and rigorous bias mitigation (random-effects modeling, subgroup analyses) enhances conclusion credibility; future research should standardize induction protocols, incorporate additional moderators (e.g., age, training experience), to address remaining limitations.

### 4.4 Study limitations

In the quality assessment of the included studies, a considerable proportion exhibited a high risk of bias in the implementation of blinding, which often stems from practical and ethical constraints in human experiments. Additionally, the diversity among subjects in terms of training background, physical fitness level, and demographic characteristics, as well as variations in PAPE induction protocols—such as exercise modality, intensity, load volume, and rest intervals—contributed to significant heterogeneity in the results, thereby limiting the generalizability of the findings. Moreover, the lack of direct physiological indicators—such as EMG and blood lactate measurements—in the existing studies restricted an in-depth interpretation of the mechanisms underlying the PAPE effect. Future research should focus on standardizing intervention protocols, enhancing physiological monitoring, and implementing subgroup analyses based on subject characteristics, along with to assess cumulative adaptive effects beyond acute potentiation.

## 5 Conclusion

PAPE may serve as a potentially effective strategy for enhancing jumping performance under appropriate conditions. In exercises aimed at improving jumping performance, back squats and medium-intensity induction appear to yield the most pronounced benefits. A 3–7 min recovery interval works best, though adjustments should be made based on individual exercise factors.

## Data Availability

The datasets presented in this study can be found in online repositories. The names of the repository/repositories and accession number(s) can be found in the article/supplementary material.
